# Quantitative Approaches to Study Retinal Neurogenesis

**DOI:** 10.3390/biomedicines9091222

**Published:** 2021-09-14

**Authors:** Diego Pérez-Dones, Mario Ledesma-Terrón, David G. Míguez

**Affiliations:** 1 Centro de Biología Molecular Severo Ochoa, Universidad Autónoma de Madrid, 28049 Madrid, Spain; diego.perezd@estudiante.uam.es (D.P.-D.); Mario.ledesma@uam.es (M.L.-T.); 2 Física de la Materia Condensada (IFIMAC), Facultad de Ciencias, Universidad Autónoma de Madrid, 28049 Madrid, Spain

**Keywords:** retinogenesis, quantitative biology, imaging

## Abstract

The study of the development of the vertebrate retina can be addressed from several perspectives: from a purely qualitative to a more quantitative approach that takes into account its spatio-temporal features, its three-dimensional structure and also the regulation and properties at the systems level. Here, we review the ongoing transition toward a full four-dimensional characterization of the developing vertebrate retina, focusing on the challenges at the experimental, image acquisition, image processing and quantification. Using the developing zebrafish retina, we illustrate how quantitative data extracted from these type of highly dense, three-dimensional tissues depend strongly on the image quality, image processing and algorithms used to segment and quantify. Therefore, we propose that the scientific community that focuses on developmental systems could strongly benefit from a more detailed disclosure of the tools and pipelines used to process and analyze images from biological samples.

## 1. Introduction

During embryo-genesis, the vast majority of the functional neurons that form the central nervous system are generated from pools of undifferentiated neural progenitor cells [1]. This process is tightly regulated at many levels by signaling pathways that are highly conserved across vertebrates. One of the main focuses in the field of developmental biology is understanding how these molecular features orchestrate neurogenesis toward the correct final form, shape, organization, and function of the organs and tissue that form the central nervous system.

Since this regulation is also highly conserved across different neurogenic tissues, the neural retina has often been used as a model system to study vertebrate neurogenesis, due mainly to its accessibility and simple structure [2,3]. The developing neural retina is a pseudostratified neuroepithelium where several major types of neurons are generated: retinal ganglion cells, cones and rods photoreceptors, amacrine, horizontal, bipolar, and Müller glia [4,5]. Cells in the developing vertebrate retina are organized in three main layers, and this structure is also highly conserved: photoreceptors localize at the outermost layer, the innermost layer is formed mostly by retinal ganglion cells, and the remaining four cell types form the inner nuclear layer (INL) [6,7].

**Figure 1:**
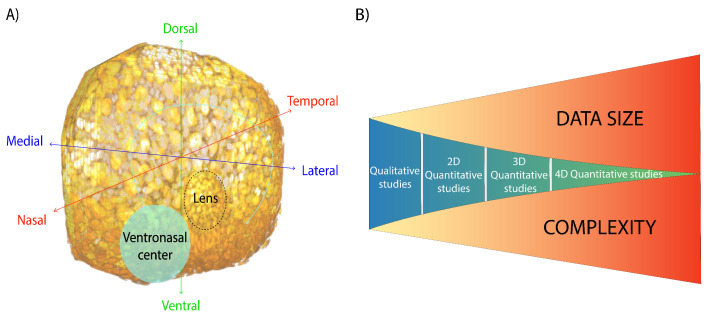
(**A**). Three-dimensional reconstruction (using FIJI plug-in 3DViewer) from confocal sections of a zebrafish retina at 44 h post fertilization. The tissue was mounted and stained with Topro3 (DNA marker) (see methods section) *in toto*. Three-dimensional reconstruction is generated. The three axes that define the developing retina are represented in three colors. (**B**). Illustration representing the shift from qualitative to quantitative studies in retinal neurogenesis. As spatial and/or temporal dimensions are taken into account, the amount of data generated and processed to obtain reliable and robust quantification increases.

From a structural and dynamic point of view, morphogenesis in the vertebrate retina follows a well-established spatio-temporal organization along three major axes (Figure 1A): dorsoventral, lateromedial and nasotemporal [8]. The sequence starts in a patch of progenitor cells, often referred to as the ventronasal center [9]. This initial group of cells starts to perform proliferative symmetric divisions, initiating a wave of proliferation that moves from central-to-peripheral and from nasal-to-temporal areas [10]. In zebrafish, this proliferative wave covers the whole embryonic retina in around 16 h [11]. Later in development, the differentiation of retinal progenitor cells (RPC) into terminally differentiated neurons also takes place as a wave, starting again at the ventronasal center and moving out toward the ventrotemporal periphery [12,13].

At the cellular level, the structure of neural retina has a marked apicobasal polarity. This asymmetrical organization of the cell membrane, the intracellular organelles and the cytoskeleton results in very important properties as well as consequences on the biochemical and even physical level. One of the most striking features is the movement of the nuclei in correlation with the cell cycle of retinal progenitors, known as interkinetic nuclear migration. During this process, cycling cells divide mostly when nuclei are localized at the apical pole, then travel to the basal zone during the G1 phase, and come back to the apical surface during G2 [14].

These multiple layers of organization, from the sub-cellular to systems levels, the sequential generation of several neuronal subtypes, the wave-like proliferation and differentiation, and the interplay between many signaling cascades, have attracted the attention of scientists from a molecular [15], cell and evolutionary biology background [16], but also researchers with a more theoretical, computational and image analysis approach [17].

Earlier studies approached the developing vertebrate retina with a focus on key aspects at the genetic and/or molecular levels, providing a highly important but mostly qualitative characterization of the processes, signals, and regulation involved in the retinal specification [18]. This basic qualitative knowledge provided the foundation for more recent contributions that studied retinogenesis from a quantitative spatial and dynamics perspective, taking advantage of more powerful and specialized tools, both at the software and hardware levels [19].

The main aim of this review is to provide a general overview of how the current understanding of neural development has benefited from studies that share a strong quantitative perspective. To do so, we organize the manuscript based of the complexity of the different approaches, focusing initially on early and recent contributions with a clear quantitative perspective, but no spatial and/or temporal information. As studies have started to incorporate dynamics or spatial analysis, the amount of data and information obtained has increased very fast, so statistical analysis, state-of-the-art imaging technology and automated segmentation methods (Figure 1B) have become an integral part of these contributions. Finally, in the last section, we propose a unified approach toward a full four-dimensional characterization of vertebrate retinogenesis.

## 2. Methods

This section describes the methodology used to produce the experimental data presented in the contribution.

### 2.1. Animals

Experiments were conducted in wild-type zebrafish embryos, sustained according to the standard procedures and protocols. All the images showing zebrafish retina images were experimentally obtained by our group. All experimental protocols are in accordance with the guidelines of the European Communities Directive (2012/63/EU) and the actual Spanish legislation (Real Decreto 53/2013).

### 2.2. Sample Preparation

Embryos are incubated in E3 1× fish medium (5 mM NaCl, 0.17 mM KCl, 0.33 mM CaCl_2_, 0.33 mM MgSO_4_) supplemented with methylene blue (Sigma, St. Louis, MO, USA) at 28 ${}^{\circ }$C. At 24 h post fertilization (*HPF*), the medium is supplemented with 0.003% phenylthiourea (Sigma, 103-85-5) to block eye pigmentation. The embryonic stage is determined by controlling the birth hour and by visual appreciation. The embryos are then fixed with a 10% formalin solution (Sigma, HT501128) by incubation overnight at 4 ${}^{\circ }$C or 2–3 h at room temperature. Finally, the embryos are washed with phosphate buffer saline (PBS) 1× three times for 5 min each.

### 2.3. Immunostaining and Mounting

First, the embryos are exposed to proteinase K (10 μg/mL; Wagen-Biotech, Los Angeles, CA, USA, 505-PKP). Different exposure times are selected according to embryonic stage of the sample, ranging from 10 (24 *HPF*) to 45 min (48 *HPF*). Then, a short fixation with 10% formalin is carried out, to prevent mechanical incidents during the experimental procedure. After the formalin solution is washed, the embryos are incubated for 1 h at room temperature or at 4 ${}^{\circ }$C overnight, with a blocking solution of fetal bovine serum (FBS) 10% in PBS 1× and 0.6% triton (PBT 0.6%) to prevent subsequent non-specific interactions. Once the blocking solution is removed, the embryos are incubated with primary antibodies diluted in a solution of FBS 2% in PBT 0.6% at 4 ${}^{\circ }$C overnight in an agitator. The following primary antibodies were used in this study to detect by immunostaining the levels of the GFP protein (1:1000; chicken, Abcam, Cambridge, UK, ab137827) and Sox2 protein (1:1000; rabbit, GeneTex, GTX124477). Finally, the embryos are incubated with secondary antibodies and other fluorescent dyes diluted in a solution of FBS 2% in PBT 0.6%. The secondary antibodies used are A-11039 from ThermoFisher, Waltham, MA, USA (1:500; chicken) and A-31573 from ThermoFisher (1:500; rabbit). The nuclei are stained with To-Pro3 (1:500; ThermoFisher, T-3605). After immunostaining, the embryos’ heads are manually dissected from their bodies, and the eyes are separated by sectioning the middle line. The embryos’ eyes are mounted in RapiClear 1.49 (SunJin Lab, Hsinchu, Taiwan) medium to minimize refraction, scattering, and dispersion.

### 2.4. Image Acquisition

Images of confocal planes for the sample (1024 × 1024 pixel resolution) were taken in a Leica SM800 confocal microscope, using a pinhole of 1 μm. An overlapping region between confocal slices of 0.2 μm was established to ensure a correct reconstruction of the tissue in the three dimensions.

Each channel was acquired separately and then, the channels were split (FIJI; Color-Split Channels) to work only with the total nuclei channel, stained with To-Pro3.

### 2.5. Object Density Analysis

To assess the density of the different focal planes presented, a small code in the Julia programming language was used. The object’s centroids were obtained from FIJI’s Analyze Particles output. Each centroid in pixels was transformed to μm by multiplying the value in pixels by the known value in microns, according to the image acquisition settings. For each object, 5 nearest neighboring objects were found based on distance from centroid to centroid. An arithmetic mean was obtained from each object, and then, the represented mean was calculated for all the objects in each image.

## 3. From Qualitative to Quantitative

As explained previously, many very important discoveries of how the early vertebrate retina is formed were based simply on qualitative observations. For instance, the determination of the main genetic and molecular signaling pathways that regulate retinal development [20], and the important identification of potential therapeutic targets within the molecules involved in these pathways [21], were based mainly on visual observation. For instance, careful *de visu* inspection and analysis were sufficient to establish that, in the zebrafish retina, the shift from progenitor to a terminally differentiated fate is mainly determined by intrinsic factors, instead of input by neighbor cells [22]. In addition, a clever qualitative approach was key to establish the role of the mouse *atonal* (*ato*) homolog *math5* in the fate of RPCs [23].

These and other similarly important studies have contributed to uncover the regulatory networks of interactions that drive the organization of retinogenesis, also showing that the molecules involved in this regulation is highly conserved in different animal model systems and tissues [24,25,26]. These scientific contributions constitute clear examples that experimental results are sometimes so evident that a careful and exhaustive quantification or statistical analysis is not essential, and sometimes does not enhance substantially the quality and integrity of the results reported.

Unfortunately, this is not always the case, and some early and recent studies that rely on just qualitative observations would strongly benefit from a more quantitative approach. For instance, studies based on visual exploration of histological sections show that the proliferation of RPCs depends critically on cyclin D1 levels since retinas appear smaller when cyclin D1 is downregulated. Based on these observations, the authors speculated that the proliferation of RPCs in the neural retina is driven by unusually high levels of cyclin D1.

In the same direction, the interaction between cyclin D1 and p27 during retinogenesis was analyzed based on visual characterization of histological sections of the developing mouse retina [27]. Another study [28] used a similar approach to conclude that the expression of cyclin D1 may alter photoreceptor cell differentiation and retina development by potentially affecting the cell cycle. Focusing also on cyclin D1, Locker et al. [24] concluded that Hh signaling decreases the expression of cyclin D1 in the *Xenopus* retina. Here, quantification of the intensity levels on microscope images of histological sections of in situ hybridization staining is presented, but the details of how these numbers are obtained are not described.

These examples of the early characterization of the developing vertebrate retina represented important contributions to the field, but they may have benefited from a more extensive quantification and analysis. In addition, a more detailed explanation of how values are obtained will arguably result in increased reliability of the data and more robust conclusions. Moreover, careful statistical analysis of the data could reveal even more relevant conclusions than simple eye inspection. For instance, in the same topic of the role of cyclin D1, the potential of a more quantitative type of approach is very well illustrated in the studies by Bienvenu et al. [29]. In this study, the authors combined microarrays, ChiP and Rt-PCR to show that in the mouse retina, cyclin D1 acts at the level of the promoters, serving as an activator and repressor of gene expression, and that reduction in the proliferation of NPCs takes place via an interplay with the Notch signaling pathway.

These types of molecular biology tools and techniques represent a step forward in terms of quantitative data, allowing us to perform statistical analysis and data processing to extract relevant information. On the other hand, they do not provide spatial and/or temporal information of how the neurogenesis in the vertebrate retina is orchestrated. As discussed in the introduction, the morphogenesis of the retina depends strongly on spatial and temporal cues; therefore, a more complete and powerful approach should take into account information about time and/or location.

## 4. 2D Quantitative Data

In the context of the developing vertebrate retina, spatial quantitative data commonly relies on features identified by in situ hybridization, immunostaining, thymidine analog incorporation, fluorescent labeling and other common techniques. Almost all of them require, at some point, the use of a microscope to visualize and register features at the subcellular, cellular or tissue levels.

Extracting quantitative data from images from biological samples can be defined as an issue that involves concepts of microscopy, computer vision and/or bioimaging. The key step to distinguish individual entities (such as cells, nuclei, organelles, etc.) in an image is segmentation, i.e., the process of separating background (noise) from foreground (information) to identify the different objects in the image [30]. Image segmentation is often regarded as the cornerstone of image analysis, and several reviews have focused on this issue in the context of biological images [31,32]. Segmentation of the objects of an image can be done manually, semi-automatically or automatically, and there are free and commercially available tools that segment images, requiring different levels of interaction and expertise by the user.

**Figure 2:**
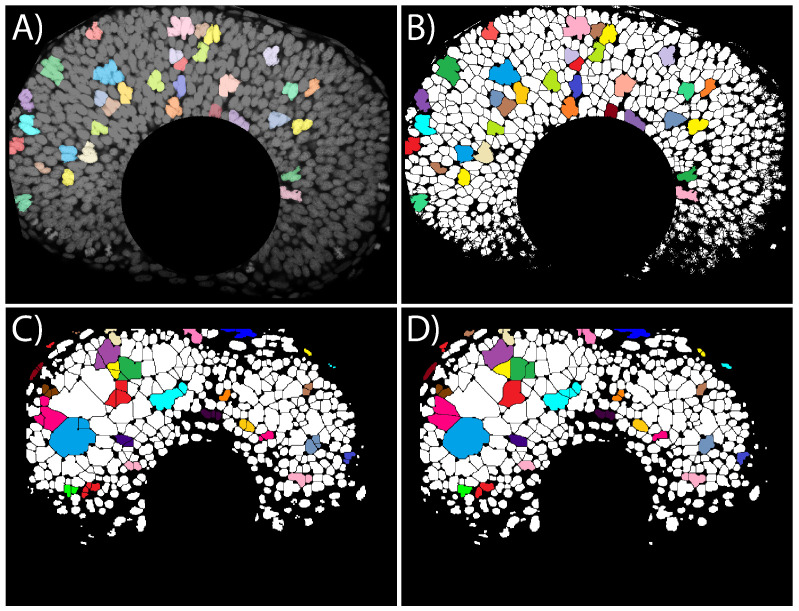
Automated segmentation of images from developing zebrafish retina. (**A**) Section of a developing zebrafish retina at 44 h post fertilization stained with nuclear marker Topro3 (see methods section). (**B**) Segmentation of the same section using Fiji base algorithms (auto-threshold + watershed). Individual objects not correctly identified are highlighted with different colors. (**C**) Image segmentation of a section of a developing zebrafish retina at 44 HPF (using FIJI base algorithms) following the pipeline: (1) automatic thresholding; (2) binary morphological opening (open function); (3) binary morphological closure (close function); (4) watershed algorithm (watershed function). (**D**) Segmentation using the exact same filters, with the only difference being that the order between closing and opening filters is reversed. Nuclei segmented differently by these two very similar pipelines are highlighted in colors.

Unfortunately, images from biological tissue, such as the developing retina, are suboptimal in terms of the signal-to-background ratio, contrast and resolution, so obtaining an accurate segmentation of the objects is not straightforward, specially when images are taken in vivo. Free and commercial general purpose tools for image segmentation are not normally designed to work in conditions of high cellular density and low contrast, and therefore, can introduce numerous segmentation errors, compromising the quantification process. To illustrate this, we show in Figure 2A a confocal section of a developing zebrafish retina at 44 h post fertilization (HPF) stained with nuclei marker and imaged in toto, using a confocal microscope. Figure 2B shows the output of a standard algorithm for automated segmentation (watershed) applied to this image. To illustrate the errors in the segmentation of the image, nuclei identified incorrectly are highlighted in color in both panels.

Due to the difficulty of accurately segmenting images from dense developing biological tissues, manual quantification is often the preferred method when working in the vertebrate retina. For instance, manual quantification is part of the pipeline used to establish the link between the mammalian homolog of inscuteable (mInsc) and the orientation of the mitotic spindle in rat retinal explants [33]. Quantitative data extraction from manual or semi-automatic analysis is also the approach used to identify the role of Sonic hedgehog (Shh) in promoting the cell–cycle exit [34] in the developing zebrafish retina, contrary to its role in promoting proliferation in other organs. In addition, the link between Shh, Gli, and Hes1 in the regulation of progenitor cell behavior [35] has been established based on manual quantification of proliferation dynamics in sections of the mouse developing retina. In addition, in the same topic of regulation of the balance between proliferation and differentiation, the role of the tumor suppressor Zac1 in promoting cell cycle exit, cell fate specification and differentiation in retinal progenitors of Xenophus [36], the spindle orientation effect in the fate of cells after division in neonatal rat retinas [37], the fate of Ath5- progenitors in zebrafish embryos using time-lapse movies [38], as well as the generation of retinal ganglion cells and photoreceptors mediated by Notch signaling [39], have been established using manual quantification.

**Figure 3:**
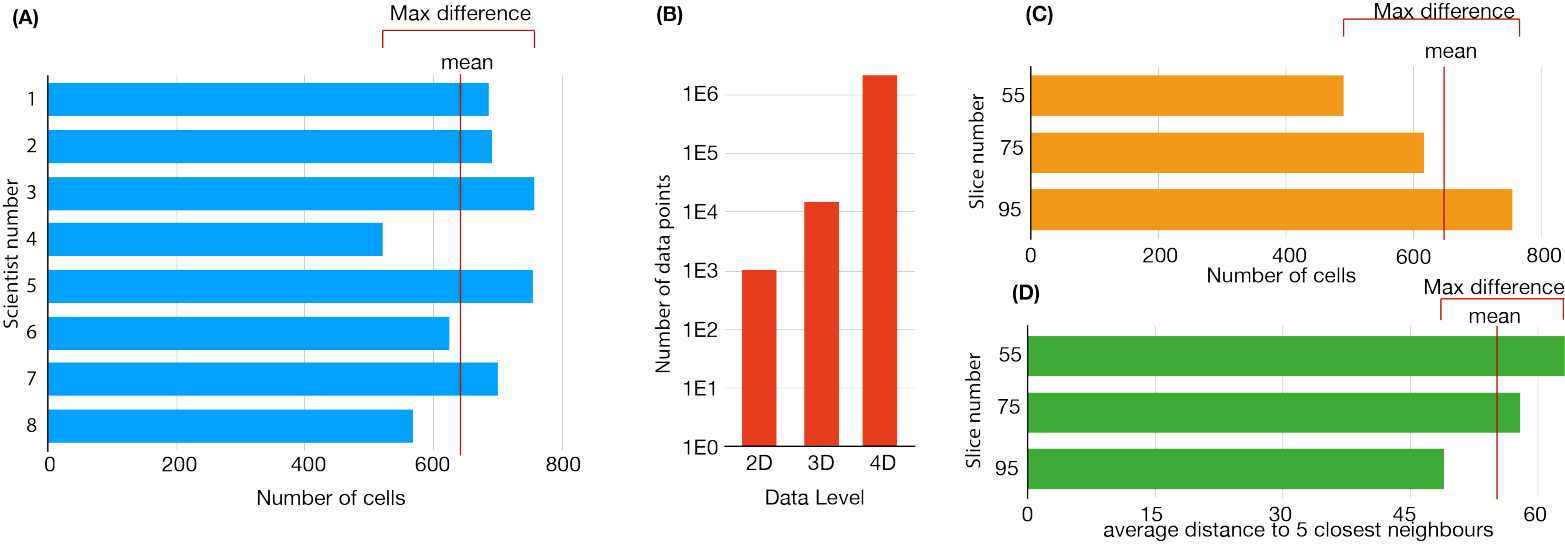
(**A**) Bar plots comparing manual quantification of the same image from trained users, using FIJI Cell Counter to assist with the quantification. Difference between users can be up to 35% of the mean value. (**B**) Increase in the number of data points to be processed when taking into account different dimensional levels of the developing vertebrate retina (vertical axis in log scale to help visualize the changes in terms of orders of magnitude). (**C**) Automated quantification of the number of nuclei of three different slices of a 3D stack of a given retina, showing differences up to 44% of the mean value. (**D**) Quantification of the nuclei density in terms of the average distance to the five closest nuclei show variations of 25% across the three different sections analyzed. Images are processed in FIJI, using an auto-threshold followed by the watershed algorithm. The number of objects is obtained from FIJI’s Analyze Particles.

The advantage of direct counting or quantification of a given output value is that it can be performed by any trained scientist with no expertise in image analysis. The main disadvantage is that it introduces a new variable in the data: the human factor. In other words, the data are inherently biased because they depend on the perception of the person; therefore, reproducibility is compromised. To illustrate this, we provided the same image of a developing zebrafish retina to a number of scientists familiar with biological images, and asked them to manually count the number of nuclei of the same section (assisted by the Cell Counter plugin from FIJI). The results (Figure 3A) show that the difference between values extracted can be up to 35% of the mean value, evidencing that *de visu* quantification, when applied to the developing retina, depends strongly on the person that performs the analysis, reducing in this way the reliability of the data.

Another limitation of manual analysis is the sample size. It is well known that the dynamics of developmental processes are very variable between individuals in the same experiment. To account for this intrinsic variability, the number of independent repeats required to obtain a reliable quantification should be substantially high (ideally, higher than the three independent repeats of a typical experimental design). Performing manual quantification becomes unattainable when using large datasets, or several independent repeats for a single data point, specially when the number of objects to count is well above three digits, as when working with mammalian retina sections.

In conclusion, manual counting might be a reasonable strategy when the images are very clear and the number of features or entities to measure is reduced. On the other hand, these are also the same conditions when direct automated segmentation performed by conventional tools produces optimal results. Therefore, when working with sections with a good signal-to-noise ratio that can be counted manually, automated image analysis should still be the preferred option because, due to its accurate performance, it reduces human bias, provides the possibility to quantify a large number of independent repeats and to perform statistical analysis to reduce the effect of variability.

## 5. Automated Image Analysis

The field of automated image processing and analysis of biological and biomedical data is very active, and many recent publications present updated tools and/or propose new methods based on state-of-the-art algorithms [19,40,41] designed to overcome the typical limitations of images from biological samples.

Careful automated image analysis was used to measure the total number of cells and retinal ganglion cells in mice [42] and rat developing retinas [43]. Additionally, the contribution of cell proliferation to the isotropic growth of the zebrafish developing retina [44], the contribution of the retinal pigmented epithelium in the formation of the optic cup [45], the role of retinoic acid [46] and the effect of ethanol exposure during retinogenenis [47,48], used automated quantitative tools.

When working with automated segmentation tools, such as the commonly used watershed or other model-based methods [49,50,51,52,53], one has to take into account that the output will depend strongly on the quality and resolution of the image. Therefore, to optimize the process of automated image segmentation and to facilitate the job of the algorithm, images are often pre-processed with a sequence of filters and transformations designed to enhance the contrast and to increase the signal-to-background ratio.

Unfortunately, this pre-processing impacts strongly the output of the segmentation, and it has to be done carefully. To illustrate this, we show in Figure 2C,D two different segmentation outputs from a section of a zebrafish retina (44 HPF, stained with nuclear marker ToPro3), using the exact same algorithms and parameters. The only difference between the left and right panels is a change in the order of application of opening and closing filters (two of the most common filters used for image processing). The large number of mismatches between the segmentation of the two almost identical pipelines (colored cells in Figure 2C,D) illustrates the importance of the pre-processing design when automatically segmenting dense images (of course, more important differences in the processing would result in larger differences).

Despite this strong impact in the quantification, the processing pipeline and/or the parameters applied are rarely described as part of the methods sections in scientific papers. One of the main reasons for this is that the pre-processing and segmentation methods in commercial tools are proprietary and, therefore, are not fully disclosed to protect the intellectual property of manufacturers. Unfortunately, this lack of transparency impacts the reproducibility (and therefore, the reliability) of the results. We strongly suggest that, similar to the detailed explanation of all experimental methodologies, reagents and protocols, image processing pipelines should be also thoroughly explained in the methods section of scientific manuscripts, including a detailed description of the filters and transformations applied, their sequence and the parameter values used. This information will ultimately facilitate other researchers to carry on similar studies.

## 6. The Developing Vertebrate Retina in Three-Dimensions

The type of quantitative analysis highlighted in the previous section focuses on comparison between still images of developing retinas at different time points or different experimental conditions. The images used are often obtained from histological sections or from single confocal planes of tissues imaged *in toto*. As explained in the introduction, the formation, growth, and specification of the vertebrate retina do not occur homogeneously in space. Several studies have reported sequential spatially asymmetric waves of proliferation and differentiation [11,12,13]. Consequently, the spatial location of a cell at a given time strongly determines its fate as a cycling progenitor or as any type of the seven differentiated neuron subtypes that compose the vertebrate retina.

For the sake of simplicity, most studies do not take into account this heterogeneity; they approach their studies focusing on representative histological sections of the developing retina. The advantage is that quasi-2D sections are easier to image, process and quantify than 3D images. The main disadvantage is that the information encoded spatially during the formation of the retina is lost when focusing on one single representative section. Moreover, the fact that differentiation and proliferation take place as a spatio-temporal three-dimensional wave makes it impossible to define what can be really considered a “representative” section of the developing retina.

On top of this, the size of the retina is also non-homogeneous, and therefore, the number of cells in the image depends on where in the retina the image was taken. To illustrate this, we show in Figure 3C three different values of the number of cells performed in three different sections of same zebrafish retina at 44 HPF (nuclei stained with ToPro3). Automated quantification of the number of nuclei shows variations of 44% of the mean value between different sections of the same 3D image. Therefore, an increase or decrease in the cell numbers associated to two different experimental conditions can be easily confused with variations in the sections being processed and quantified. Quantification of the cell density also shows variations between the planes (Figure 3D); this is important because it directly affects the performance of the segmentation algorithms, resulting in important changes in the accuracy of the data depending on the section selected. Based on this, we suggest that the optimal way to correctly quantify a heterogeneous tissue, such as the developing retina, is a three-dimensional approach.

The shift from 2D to 3D in many areas of biosciences and biomedical research is already in progress, taking advantage of improved hardware and software tools [54]. At the experimental level, one of the main problems when imaging thick whole-mount three-dimensional tissues is the absorption and scattering of photons when traveling through the sample. This results in blurred, noisier images as we focus on regions deep into the organ. Fortunately, this can be minimized in vitro by using mounting solutions that improve the transparency of biological tissues [55]. These mounting solutions or treatments can reduce the differences in the refractive index (RI) between the tissue and the surrounding medium. Additionally, they can modify the size and structure of biological molecules responsible for light scattering, such as collagen [56,57]. Finally, tissue dehydration may be also contributing to the clearing process, affecting the density and molecular organization, and therefore, decreasing light scattering [58].

To minimize loss of resolution due to light absorption, chemical treatment is often applied to decolorize the tissue. In the context of the developing retina, melanin is one of the main molecules that absorb light. In zebrafish and other organisms, phenylthiourea (PTU) is often added to the media where animals are developing to inhibit myelinization and reduce the opacity of the tissue, although it may have side effects in other aspects of the developmental processes [59].

From the hardware perspective, several recent tools have been developed and improved that facilitate the acquisition of data and image from large dense samples with quantitative quality. Conventional or two-photon confocal microscopy can be used to perform 3D optical sectioning on whole mount tissues. One of their main limitations is speed because images are registered based on point-scanning (i.e., voxel-by-voxel). This increases the exposure of the sample to a highly energetic laser to cover all confocal sections of a thick sample, resulting in increased photobleaching and photo-toxicity that can damage the tissue and affect the quality of the image [60].

Due to these limitations, light-sheet fluorescence microscopy (LSFM) arises as the optimal approach when working with thick 3D samples. In these systems, excitation light and emission light are emitted and captured, respectively, by two different lenses, while the sample or the light source rotates to image the whole tissue.

This way, instead of the point-scanning method of confocal microscopy, a whole section can be imaged and registered at once, resulting in a much higher acquisition speed. Additionally, by using two objectives instead of one, photons only have to travel through the sample once, minimizing photobleaching, phototoxicity, light absorption and light dispersion [61].

In the context of vertebrate retinogenesis, LSFM has been already used to reconstruct the early development of zebrafish embryos at a speed of 1.5 billion voxels per minute to study global cell division patterns [62]. Additionally, elective plane illumination microscopy (SPIM) has been used to study characteristic migration patterns and global tissue remodeling in the early endoderm [63] of zebrafish embryos.

After image acquisition, the next challenge is the quantification of these 3D images. Three-dimensional reconstructions, either from confocal or LSFM, can illustrate very beautifully the shape and organization of the tissue, but translating all this information into numbers is virtually impossible without the help of specialized software. Several commercial and open-source computational tools have been developed and are widely used in this context. Again, similar to what occurs in two-dimensions, the output of the quantification depends strongly on the quality of the image, the processing pipeline, the parameters, and the type of algorithm for segmentation as well as, ultimately, the software used.

Different computational tools produce different output because they rely on different approaches, from conventional mathematical operations designed to filter and segment objects, to state-of-the-art deep learning neural networks [64] that identify objects based on hundreds of features or rules. Some deep-learning implementations are focused on tasks such as object detection [65,66], image segmentation [67,68], object tracking [69,70], object classification [71] or a combination of these [72,73,74,75,76].

Due to this core differences, a given algorithm or tool may work better in one type of three-dimensional image than others. Additionally, these tools were mainly designed to quantify images with large empty spaces between objects, or a very good signal-to-background ratio. Unfortunately, images from three-dimensional dense biological tissues, such as the developing vertebrate retina, often have a resolution and contrast that is far from optimal, specially when imaged in vivo. In these conditions, conventional tools do not produce accurate results, so the full potential of a three-dimensional approach can be compromised by the lack of reliable quantification tools.

**Figure 4:**
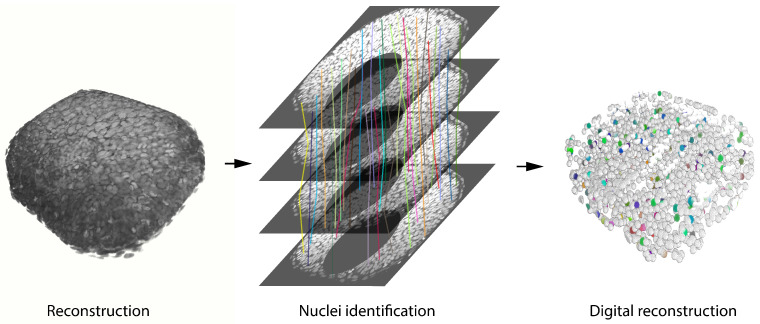
Schematic representation of the pipeline followed to reconstruct objects in three-dimensional images by OSCAR. (**Left**): 3D representation of the original image (FIJI-3D Viewer). (**Center**): representation of how neighboring sections from the same 3D object are connected. Each colored line follows the trajectory of a given nucleus along the z-*axis*. (**Right**): 3D representation of the reconstructed 3D image. Colored objects represent the nuclei that correspond to the colored lines in the central panel. A processing and segmentation pipeline is applied to the original image in order to obtain a more reliable reconstruction (Gaussian blur sigma = 20 pix; Minimum filter sigma = 20 pix; Maximum filter sigma = 20 pix; Image Calculator—Original image subtract filtered; Manual thresholding; Binary Open; Watershed; 3D Objects Counter; 3D Draw Shape—All 3D objects represented as 10 pix radius spheres; 3D Viewer).

In this direction, we have recently developed OSCAR: an object segmentation counter and analysis resource that is designed to work with three-dimensional images where the resolution is low and/or the object density is high [19]. Our tool combines nonlinear fitting algorithms with statistical analysis to bypass segmentation errors that frequently take place when segmenting automatically low-resolution images. In brief, OSCAR reconstructs three-dimensional objects by taking advantage of the three-dimensional information in the image and correct the mistakes that may occur in the segmentation process. An illustration of the process performed by OSCAR is shown in Figure 4. The three-dimensional image is processed, filtered, enhanced and segmented in a plane-by-plane basis. Next, objects identified in neighboring planes are connected based on statistical analysis and nonlinear fitting algorithms. Finally, a digital representation is generated based on the geometry of the objects detected. Our results show that OSCAR is able to outperform other tools used commonly for image analysis in conditions of low resolution and low signal-to-background, typical of biological 3D images registered in toto or in vivo [19].

## 7. The Developing Vertebrate Retina in Four-Dimensions

Neurogenesis is a highly dynamic process with many signals and events that depend on time. Before neurogenesis starts, neural stem cells are organized in a single pseudostratified layer of neuroepithelial cells [45,77]. After the formation of the primordium of the central nervous system as a neural tube, two protuberances appear at the sides of the future brain, called optic vesicles (lobes in zebrafish). Later on, these regions differentiate into the neural retina, the retinal pigmented epithelium (RPE) and the optic cup. Finally, the interplay between the formation of the RPE and the invagination of the optic lobe ultimately shapes the whole optic cup [78,79].

Another very direct indication that illustrates how the core features of retinogenesis depend on time is the changes in the differentiation probability of the retinal progenitor cells into the different subtypes of terminally differentiated neurons. These type of decisions have been shown to follow a stochastic pattern in the vertebrate developing retina, with the probabilities of differentiation occurring in a well conserved sequential order [80,81]: the initial wave of differentiation into retinal ganglion cells is followed by a second wave in which the horizontal cells and cones are originated, which then partially overlap in time with the following wave of retinogenesis, giving rise to amacrine cells, while also overlapping with the differentiation stage into rods and, during a shorter period, bipolar cells. Finally, a last wave of differentiation originates Müller glia [80,81,82].

Interkinetic nuclear migration (INM) is another very important process with a strong dynamic component. This coupling between nuclei displacement and cell cycle progression is driven mainly by forces acting at the cytoskeleton level; the causes and consequences of INM are still being elucidated [83].

These three illustrations of highly important dynamic processes that shape the growth, formation, and specification of the vertebrate retina suggest that a full quantitative characterization also has to take into account the temporal variable.

To date, this type of full four-dimensional characterization is not available for the vertebrate developing retina. On the other hand, it has been successfully implemented for segmenting 4D images of cardiac magnetic resonances [84] for assessing the whole embryonic development of the zebrafish in the first 24 h [85], for establishing the trichome patterning of *Arabidopsis thaliana* through 4D confocal images [86] or for quantitatively analyzing 4D confocal images of anchor-cell invasion in *Caenorhabditis elegans* [87].

Due to the already-mentioned accessibility of the retina, we believe that a full four-dimensional characterization of its development at single cell resolution is not beyond reach. An even more powerful approach will be to perform these type of time-lapse movies in animals where the fate of the cells can be identified in vivo, using fluorescent reporters of differentiation, such as the widely used zebrafish ath5:GFP transgenic line (engineered to express the green fluorescent protein (GFP) under regulation of the promoter of the Atonal-homolog 5 protein (ath5) [38], the Fucci system to monitor cell cycle progression [88,89] or fluorescent reporters of the mode of division [90]. A combination of a four-dimensional approach with these type of molecular biology tools would provide us with the ultimate weapon to fully characterize the developing vertebrate retina.

## 8. Discussion

The vertebrate developing retina is an optimal model system to study the dynamics and the balance between proliferation and differentiation during neurogenesis, both at single cell resolution and at the system level. Its size and accessibility allows us to obtain in toto images and time-lapse movies with quantitative detail. Combined with antibody staining or fluorescent reporters to discriminate between the different cell fates that coexist in its pseudostratified organization, it is an ideal candidate system for multi-level spatial and temporal approaches.

After the recent advances in microscopy techniques, such as the already-mentioned LSFM technology, the bottleneck toward this full characterization of vertebrate retinal development is the availability of fast, automatic and reliable methods of 3D image segmentation and cell tracking. Automated cell tracking of the position and fate of all the cells in a three-dimensional tissue as dense as the developing vertebrate retina is a very challenging and demanding task.

In addition, this approach involves the additional challenge of processing of a large amount of information. For instance, in the context of the developing zebrafish retina, a minimum of a 24 h period is needed to account for the complete developmental process of the first wave of differentiation of neurons that form the retina. Assuming a rather conservative 10 min spacing between frames, a single time-lapse movie should contain a minimum of 144 frames. From each time frame, a minimum of 100 slices are needed for a proper 3D reconstruction of a single retina. As a result, a minimum of 14,400 slices have to be segmented and processed. The typical three independent repeats for a given experiment will result in more than 40,000 2D images for a single experimental condition.

At the cellular level, the position of each cell in the 3D space has to be processed to connect objects in consecutive frames. Assuming that a zebrafish retina, if formed by around 5000 at the time that the first wave of differentiation finishes (at around 44 HPF), tracking all the cells over 24 h will require the processing of above 2e6 data points (three spatial coordinates per cell). Figure 3B shows an estimation of how the number of the data increases as we take into account more dimensions, with an increase of one order of magnitude from 2D sections to the 3D full tissue. The data processed increase by two extra orders of magnitude when the temporal dimension is added.

Once this type of tool is available and optimized, the next step is the comparison of homeostatic conditions with situations where some signaling pathways are disrupted by small molecule inhibition. Quantification of the differences in the mode and/or rate of division will allow us to unveil the true role of each signaling network, and how some of them interplay during the orchestration of the multiple spatial and temporal processes that result in a fully functional retina.

## 9. Conclusions

The field of developmental biology, and, in particular, retinal neurogenesis, has become an example of good practices when studying heterogeneous tree-dimensional tissues from a quantitative perspective with accurate spatial and temporal resolutions. To move further into this direction, an increased effort should be focused into describing with sufficient detail the pipelines and tools used in the acquisition, processing, quantification, and data analysis. We hope that the present analysis of the state of the art in the field illustrates that a full multi-dimensional quantitative characterization of the developing vertebrate retina can only be achieved as a collective effort.

## Data Availability

Data are available on request by the corresponding author.

## Sample Availability

Samples of the compounds, and code used are available from the authors.

## Abbreviations

The following abbreviations are used in this manuscript:

RPC	Retinal progenitor cell
HPF	Hours post fertilization
mInsc	Mammalian homolog of Inscuteable
LSFM	Light sheet fluorescence microscopy
SPIM	Selective plane illumination microscopy
iPS	Induced pluripotent stem
